# Long-term all-cause death prediction by coronary, aortic, and valvular calcification in patients with acute ST-segment elevation myocardial infarction

**DOI:** 10.1186/s12872-024-03758-6

**Published:** 2024-02-19

**Authors:** Yalin Cheng, Xuyang Meng, Haiyang Gao, Chenguang Yang, Peng Li, Hongfei Li, Saurav Chatterjee, Paulo Cury Rezende, Marc Bonnet, Huimin Li, Zunlei Zhang, Fusui Ji, Wenduo Zhang

**Affiliations:** 1 Department of Cardiology, Beijing Hospital, National Center of Gerontology, Institute of Geriatric Medicine, Chinese Academy of Medical Sciences, Beijing, 100730 China; 2 Clinical Assistant Professor of Medicine, Northwell Health, Zucker School of Medicine, Hempstead, NY USA; 3 Instituto do Coração (InCor), Hospital das Clínicas, Faculdade de Medicina, Universidade de São Paulo, São Paulo, Brazil; 4 Cardiology Department, Hospital of Annecy, Annecy, France; 5 Department of Cardiology, People’s Hospital of Weishan County, Jining, Shandong 277600 China

**Keywords:** Myocardial infarction, Calcification, Risk score, Prognosis

## Abstract

**Background:**

To determine the prognostic value of cumulative calcification score of coronary artery calcification (CAC), thoracic aortic calcification (TAC) and aortic valve calcification (AVC) in acute ST segment elevation myocardial infarction (STEMI) patients.

**Methods:**

This was a retrospective, single-center cohort study. A total of 332 STEMI patients who received primary percutaneous coronary intervention (PPCI) were enrolled in this study between January 2010 to October 2018. We assessed the calcification in the left anterior descending branch (LAD), left circumflex branch (LCX), right coronary artery (RCA), thoracic aorta, and aortic valve. Calcification of each part was counted as 1 point, and the cumulative calcification score was calculated as the sum of all points. The primary endpoint was all-cause mortality. Multivariate Cox proportional hazards models were used to determine association of cumulative calcification score with end points. The performance of the score was evaluated by receiver operating characteristic (ROC) curve analysis and absolute net reclassification improvement (NRI), compared with the Global Registry of Acute Coronary Events (GRACE) risk score.

**Results:**

The overall population’s calcification score was 2.0 ± 1.6. During a mean follow-up time of 69.8 ± 29.3 months, the all-cause mortality rate was 12.1%. Kaplan-Meier curve showed that the score was significantly associated with mortality (log-rank *p* < 0.001). The multivariable Cox proportional hazard analyses showed that a calcification score of 4–5 was independently associated with all-cause death in STEMI patients [hazard ratio (HR) = 2.32, 95% confidence interval (CI): 1.01–5.31, *p* = 0.046]. The area under the ROC curve (AUC) of the calcification score was 0.67 (95% CI: 0.61–0.72), and the AUC of the GRACE score was 0.80 (95% CI: 0.75–0.84). There was no statistical difference in the predictive value between both scores for 3-year mortality in STEMI patients after PPCI (*p* = 0.06). Based on the NRI analysis, the calcification score showed better risk classification compared with the GRACE score (absolute NRI = 6.63%, *P* = 0.027).

**Conclusion:**

The cumulative calcification score is independently associated with the long-term prognosis of STEMI patients after PPCI.

## Introduction

Vascular calcification—pathological deposition of hydroxyapatite crystals—can occur throughout the vascular system, including large arteries such as the aorta, carotids, and tibial arteries, as well as in smaller vessels such as coronary arteries and skin capillaries. Coronary artery calcification (CAC), as an important manifestation of subclinical atherosclerosis, is associated with the future cardiovascular disease outcomes and can be used for informing the treatment decision-making for preventive therapies [1, 2].

Non-coronary vascular calcifications including the thoracic aortic calcification (TAC) and aortic valve calcification (AVC) are also associated with the increased risk for adverse cardiovascular events and mortality [3–5].

However, the value of prediction and risk reclassification by cumulative calcification score of CAC, TAC, and AVC in acute ST segment elevation myocardial infarction (STEMI) patients remains unclear.

In this study, we calculated the cumulative calcification scores of CAC, TAC, and AVC, and explored the predictive value of the scores for long-term prognosis in acute STEMI patients.

## Methods

### Study population

**Fig. 1:**
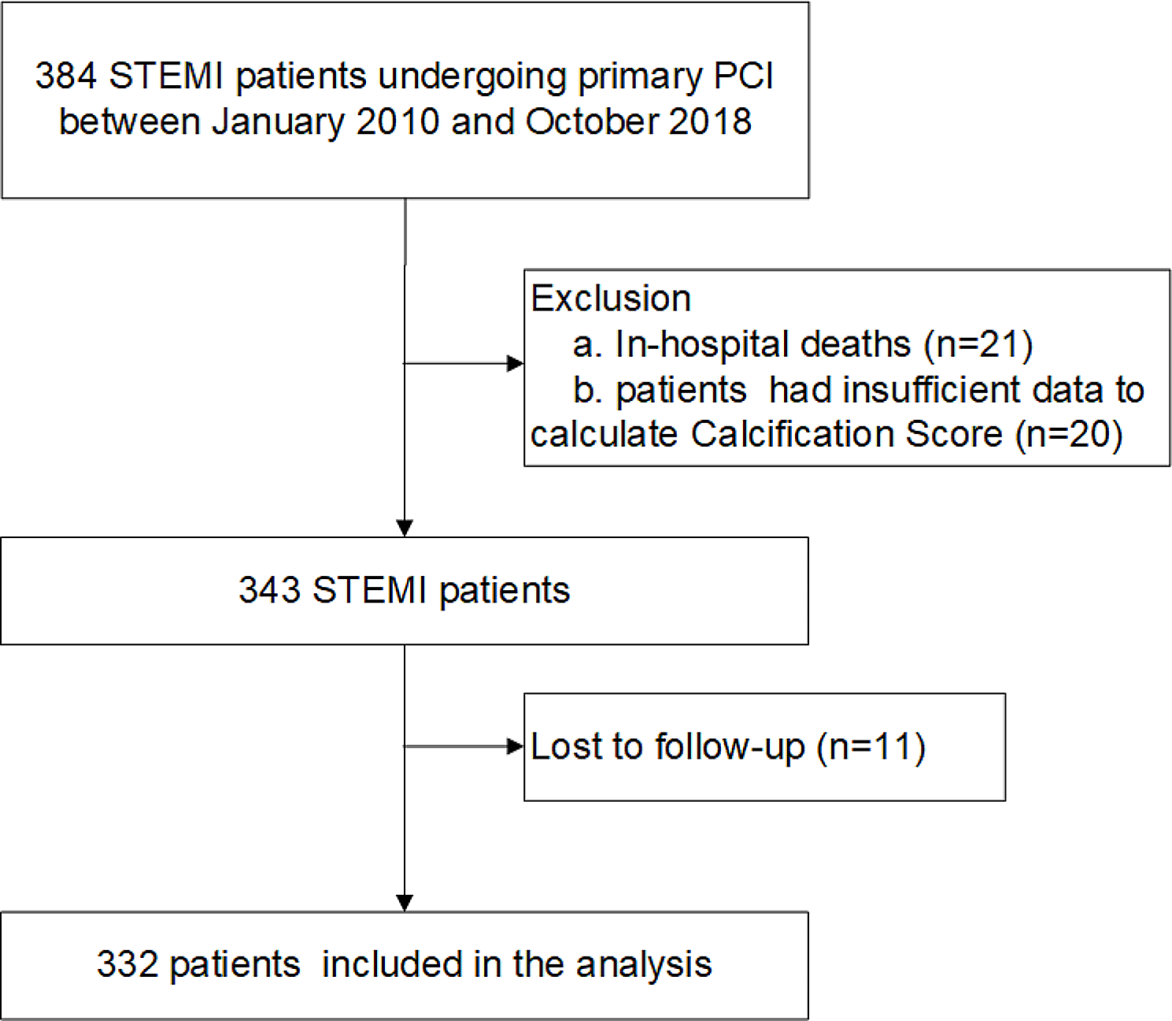
Patient flowchart of this study

This was a retrospective observational study. Patients with STEMI who received primary percutaneous coronary intervention (PPCI) in Beijing Hospital from January 2010 to October 2018 were enrolled consecutively. The patient flowchart is shown in Fig. 1. STEMI diagnosis was determined by experienced cardiologists using the 2017 ESC Guidelines for the Diagnosis and Treatment of Acute ST-segment Elevation Myocardial Infarction [6]: myocardial markers are higher than the upper limit of the reference value and there is at least 1 indication of myocardial ischemia as follows: (I) clinical symptoms of myocardial ischemia; (II) ST segment elevation ≥ 0.1 mV in 2 or more adjacent leads of electrocardiogram (ECG), new left bundle branch block or pathological Q wave; (III) imaging evidence of a new myocardial loss or regional wall motion abnormality. We included the STEMI patients who received PPCI treatment with symptom onset-to-door time < 12 h or ≥ 12 h but < 24 h accompanied by persistent unrelieved chest pain, ECG dynamic changes, and hemodynamic instability. The exclusion criteria were as follows: (I) in-hospital death; (II) insufficient data to calculate the calcification score; (III) coronary artery bypass grafting; (IV) valve replacement. All participants signed the informed consent form. This study conformed to the Declaration of Helsinki (as revised in 2013) and was approved by the Ethics Committee of Beijing Hospital (No. 2016BJYYEC-121-02). The clinical data of each patient including demographic, risk factors, Killip classification, the Global Registry of Acute Coronary Events (GRACE) risk score, laboratory data, echocardiographic data, and angiographic data were collected from the electronic medical records by professionally trained doctors.

### Cumulative calcification scores

In this study, the calcifications in coronary artery, thoracic aorta, and aortic valve were assessed by 2 physicians blinded to the outcomes of all patients.

**Fig. 2:**
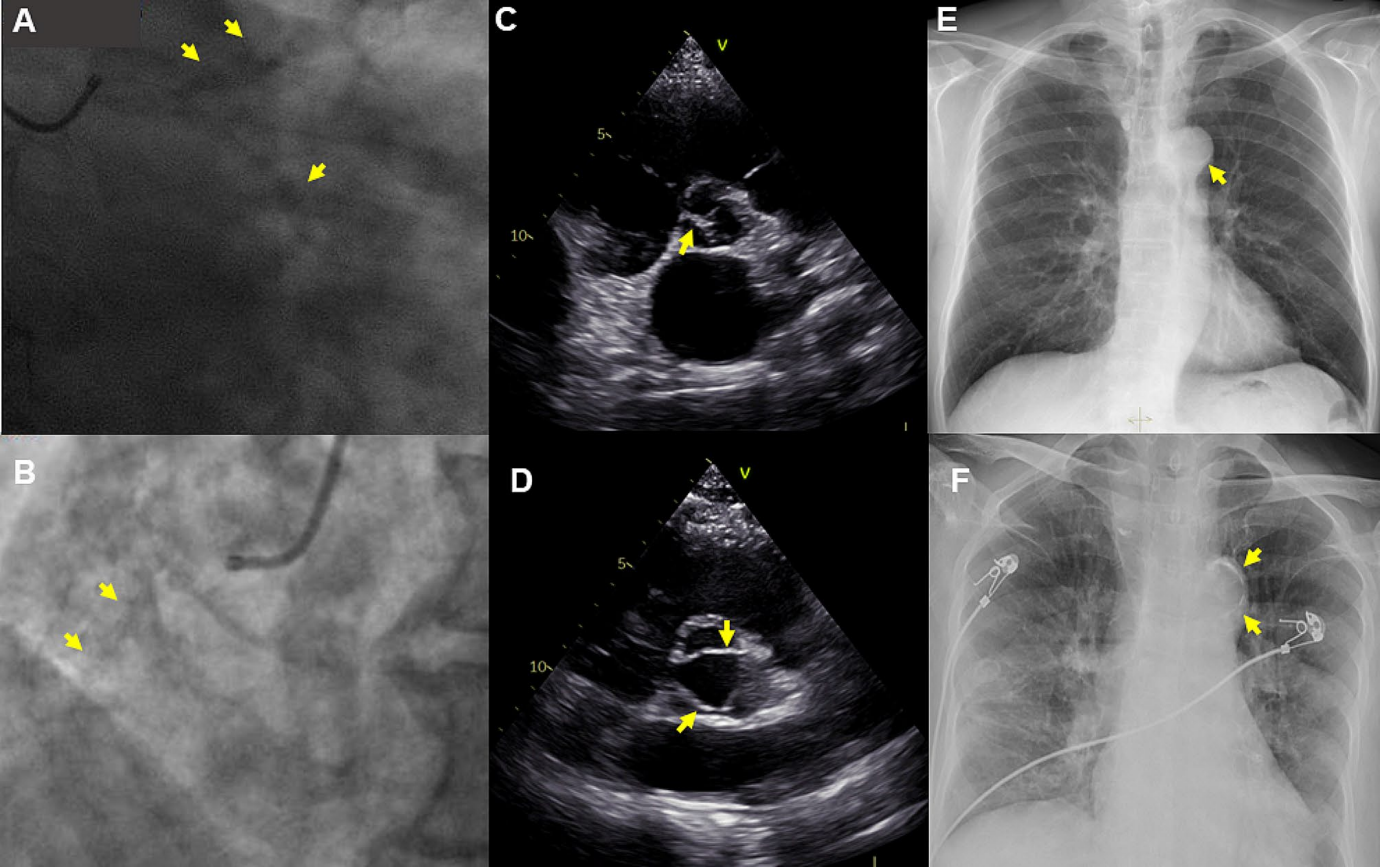
(**A** and **B**) Coronary artery calcification (CAC) in coronary angiography. (C and D) Aortic valve calcification (AVC) in echocardiography. (**E** and **F**) Thoracic aortic calcification (TAC) in chest X-ray

For CAC, the calcifications in left anterior descending branch (LAD), left circumflex branch (LCX), and right coronary artery (RCA) were evaluated using invasive coronary angiography (ICA). The definition of calcification was radiopaque densities involving one or two sides of the vascular wall (Fig. 2A, B) [7]. The ICA was assessed by two experienced cardiologists to identify CAC. They didn’t know the TAC, AVC, or the outcomes. TAC was assessed based on the presence of visible calcification in thoracic aortic on chest X-rays (DXR-Revolution: Carestream Health, Rochester, NY, USA) by two radiologists (Fig. 2C, D) [3]. AVC was evaluated by echocardiography which was conducted and analyzed by cardiologists specializing in cardiac imaging (Fig. 2E, F) [5].

We assessed the calcification in LAD, LCX, RCA, thoracic aorta, and aortic valve. Calcification of each part was counted as 1 point, and the total calcification score was calculated as the sum of all points. Participants scoring 0–1 point were allocated to the low calcification degree group (group 1), 2–3 points into the medium calcification degree group (group 2), and 4–5 into the high calcification degree group (group 3).

### Follow-up and outcomes

The primary endpoint was all-cause mortality. The survival status was ascertained by telephone interview, examination of hospital records, outpatient department, or death certificates.

### Statistical analysis

The characteristics of all patients at baseline were presented as means with standard deviations (SDs) for continuous variables that were normally distributed, median (interquartile range) for continuous variables without normal distribution, and number (percentage) for categorical variables. One-way analysis of variance (ANOVA) was used for comparisons of continuous variables between groups, and the χ^2^ test was used for categorical variables. Bonferroni correction was used for multiple comparisons of the three groups. Survival analysis according to scores was carried out by the Kaplan-Meier method, and comparisons were made using the log-rank test. Cox regression was used to examine the associations of calcification score with all-cause mortality. Parameters with statistical significance in the univariable analysis (*p* < 0.05) were included in the multivariable analysis. Receiver operating characteristic (ROC) curves and the area under the ROC curve (AUC) with 95% confidence interval (CI) were calculated to compare the predictive ability of calcification score and GRACE risk score for 3-year mortality. To assess the global performance of the two scores, absolute net reclassification improvement (NRI) was used.

ROC curves were performed by MedCalc software v 18.2.1 (MedCalc Software, Mariakerke, Belgium). Other analyses were performed using SPSS v 23.0 (IBM Corp., Armonk, NY, USA) and GraphPad Prism 8 (GraphPad, San Diego, CA, USA). A 2-sided *p* < 0.05 was considered significan.

## Results

### Baseline characteristics

A total of 332 STEMI patients were enrolled in this study. The characteristics of the study population are listed in Table 1. The mean age was 62.9 years and male patients accounted for 76.2%. Of these STEMI patients, 59.6%, 52.6%, and 32.3% had hypertension, dyslipidemia, and diabetes mellitus, respectively. At admission, 7.3% of the patients had Killip classification III–IV and the mean GRACE risk score was 156.9 ± 32.0. According to the calcification score, there were 148 cases in group 1, 111 cases in group 2, and 73 cases in group 3. With the score increasing, patients were older, more often of the female sex, and had more risk factors such as hypertension and diabetes mellitus. In the higher calcification group, GRACE risk score, and brain natriuretic peptide (BNP) levels were higher (Table 1).

**Table 1:** Comparisons of clinical characteristics among the three calcification score groups

Variable	Overall (*n* = 332)	Group 1 (*n* = 148)	Group 2 (*n* = 111)	Group 3 (*n* = 73)	*P* value
**Demographics**					
Age, years	62.9 ± 12.3	56.2 ± 10.4	65.6 ± 11.1^*^	72.1 ± 10.1^*§^	**< 0.001**
Male, n (%)	253 (76.2)	131 (88.5)	84 (75.7) ^*^	38 (52.1) ^*§^	**< 0.001**
BMI, kg/m^2^	24.9 ± 3.3	25.4 ± 2.8	24.9 ± 3.3	24.0 ± 3.9^*^	**0.009**
**Risk factors, n (%)**					
Hypertension	198 (59.6)	74 (50.0)	74 (66.7) ^*^	50 (68.5) ^*^	**0.006**
Dyslipidemia	174 (52.6)	78 (52.7)	62 (56.4)	34 (46.6)	0.43
Diabetes mellitus	107 (32.3)	37 (25.0)	36 (32.4) ^*^	34 (46.6) ^*^	**0.006**
Current smokers	203 (61.1)	109 (73.7)	64 (57.7) ^*^	30 (41.1) ^*^	**< 0.001**
Family history of premature CHD	56 (16.9)	32 (21.6)	18 (16.4) ^*^	6 (8.2) ^*^	**0.032**
Previous MI	16 (4.8)	9 (6.1)	4 (3.6)	3 (4.1)	0.63
Previous PCI	34 (10.3)	17 (11.5)	11 (10.0)	6 (8.2)	0.74
**Killip classification, n (%)**					0.073
I	243 (73.9)	117 (80.1)	81 (73.6)	45 (61.6)	
II	62 (18.8)	22 (15.1)	19 (17.3)	21 (28.8)	
III	11 (3.3)	4 (2.7)	3 (2.7)	4 (5.5)	
VI	13 (4.0)	3 (2.1)	7 (6.4)	3 (4.1)	
**GRACE risk score**	156.9 ± 32.0	143.7 ± 25.8	108.9 ± 33.4^*^	174.0 ± 30.3^*§^	**< 0.001**
**Lab results**					
TC, mmol/L	4.2 ± 1.1	4.3 ± 1.2	4.3 ± 1.0	4.2 ± 1.0	0.94
TG, mmol/L	1.7 ± 1.4	1.8 ± 1.5	1.7 ± 1.3	1.5 ± 1.1	0.18
LDL-c, mmol/L	2.7 ± 0.9	2.7 ± 0.9	2.7 ± 0.8	2.7 ± 0.9	0.98
HDL-c, mmol/L	1.0 ± 0.2	1.0 ± 0.2	1.0 ± 0.2	1.0 ± 0.2	0.13
FBG, mmol/L	7.5 ± 3.8	7.3 ± 4.5	7.8 ± 3.5	7.8 ± 2.8	0.56
HbA1c, %	7.1 ± 1.7	7.4 ± 1.8	6.6 ± 1.3	7.1 ± 2.0	0.18
Uric acid, mmol/L	332.3 ± 97.8	341.3 ± 98.1	327.4 ± 100.8	321.3 ± 92.2	0.29
Serum creatinine, µmol/L	72.0 (63.0, 84.0)	72.0 (63.3, 82.8)	73.0 (64.0, 87.5)	71.0 (55.5, 84.5)	0.26
BNP, pg/mL	205.3 (88.2, 393.8)	161.0 (72.6, 236.6)	138.8 (81.7, 693.5)	375.2 (232.9, 563.3) ^*^	**0.008**
**Echocardiographic data**					
LVEDD, mm	47.7 ± 5.2	48.4 ± 5.3	47.2 ± 5.2	46.9 ± 4.8	0.069
LAD, mm	36.3 ± 4.3	36.1 ± 4.0	36.4 ± 4.8	36.7 ± 3.9	0.6
LVEF, %	50.1 ± 12.2	50.9 ± 1.0	49.4 ± 1.2	49.5 ± 1.4	0.55
**Vascular and valvular calcifications**					
Coronary artery calcification, n (%)					
Left anterior descending branch	170 (51.2)	18 (12.2)	79 (71.2) ^*^	73 (100) ^*§^	**< 0.001**
Left circumflex artery	84 (25.3)	0 (0)	20 (18.0) ^*^	64 (87.7) ^*§^	**< 0.001**
Right coronary artery	105 (31.6)	6 (4.0)	33 (29.7) ^*^	66 (90.4) ^*§^	**< 0.001**
Thoracic aorta calcification, n (%)	135 (40.7)	18 (12.2)	63 (56.8) ^*^	54 (74.0) ^*^	**< 0.001**
Aortic valve calcification, n (%)	191 (57.5)	43 (29.0)	81 (73.0) ^*^	67 (91.8) ^*§^	**< 0.001**
Calcification score	2.0 ± 1.6	0.6 ± 0.5	2.5 ± 0.5^*^	4.4 ± 0.5^*§^	**< 0.001**

Values are mean ± standard deviation, n (%), or median (interquartile range). *BMI* body mass index, *CHD* coronary heart disease, *MI* myocardial infarction, *PCI* percutaneous coronary intervention, *TC* total cholesterol, *TG* triglyceride, *LDL-c* low-density lipoprotein cholesterol, *HDL-c* high-density lipoprotein cholesterol, *FBG* fasting blood glucose, *BNP* brain natriuretic peptide, *LVEDD* left ventricular end-diastolic diameter, *LAD* left atrium diameter, *LVEF* left ventricular ejection fraction

^*^P < 0.05 vs. Group 1

^§^*P* < 0.05 vs. Group 2

The overall population’s calcification score was 2.0 ± 1.6, of which 51.2% had LAD artery calcification, 25.3% had LCX calcification, 31.6% had RCA calcification, 57.5% had TAC, and 40.7% had ACV. With the increase of calcification score, the proportion of patients with 3 lesions and culprit vessels of the RCA increased (Tables 1 and 2).

**Table 2:** Angiographic findings and all-cause death during follow-up among the three calcification score groups

Variable	Overall (*n* = 332)	Group 1 (*n* = 148)	Group 2 (*n* = 111)	Group 3 (*n* = 73)	*P* value
**CAD severity, n (%)**					**< 0.001**
One-vessel disease	72 (21.8)	50 (33.8)	19 (17.3) ^*^	3 (4.1) ^*§^	
Two-vessel disease	107 (32.3)	52 (35.1)	34 (30.9)	21 (28.8)	
Three-vessel or LM disease	152 (45.9)	46 (31.1)	57 (51.8) ^*^	49 (67.1) ^*^	
**Culprit vessel, n (%)**					**0.033**
LM	1 (0.3)	0 (0)	0 (0)	1 (1.4)	
LAD	176 (53.2)	84 (56.8)	61 (55.5)	31 (42.5)	
LCX	43 (13.0)	25 (16.9)	10 (9.1)	8 (11.0)	
RCA	111 (33.5)	39 (26.4)	39 (35.4) ^*^	33 (45.2) ^*^	
TIMI flow 0	216 (65.5)	102 (68.9)	72 (65.5)	42 (58.3)	0.30
Collateral circulation	78 (24.9)	37 (26.6)	25 (23.4)	16 (22.9)	0.78
Proximal lesion	115 (35.0)	46 (31.3)	43 (39.1)	26 (36.1)	0.42
**Median follow-up time, months**	69.8 ± 29.3	76.3 ± 28.8	68.1 ± 29.3	59.4 ± 27.4	**< 0.001**

Values are mean ± standard deviation or n (%). *CAD* coronary artery disease, *LAD* left anterior descending branch, *LM* left main coronary artery, *LCX* left circumflex artery, *RCA* right coronary artery

^*^P < 0.05 vs. Group 1

^§^*P* < 0.05 vs. Group 2

### Inter-observer variability

Diagnosis of CAC by ICA was well reproducible [concordance: positive and negative scans 99% (357/359 and 679/677, respectively). Diagnosis of TAC by X-rays was well reproducible [concordance: positive and negative scans 96% (130/135 and 202/197, respectively). Diagnosis of AVC by echocardiography was well reproducible [concordance: positive and negative scans 96% (185/191 and 147/141, respectively).

### Clinical outcomes

**Fig. 3:**
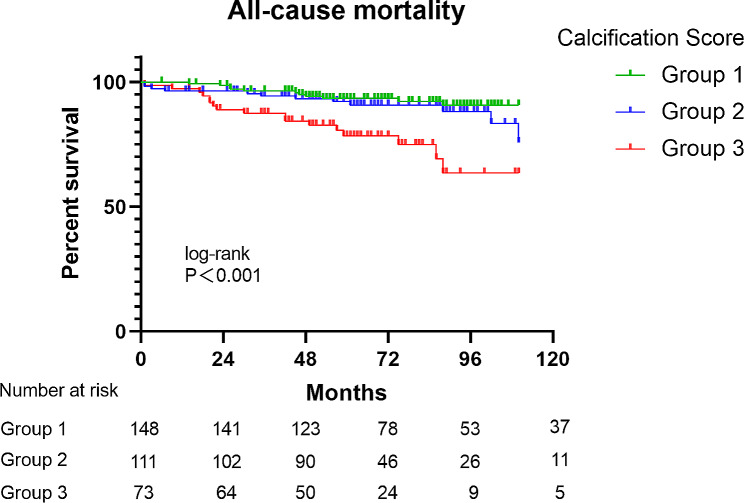
Kaplan-Meier curves of all-cause mortality stratified by calcification score. The score was significantly associated with mortality (log-rank *p* < 0.001)

The follow-up results are shown in Table 2. During a mean follow-up time of 69.8 ± 29.3 months, the all-cause mortality rate was 12.1%. With the increase of calcification fraction, all-cause mortality in group 3 was significantly higher compared with group 1 and 2 (*p* = 0.003). Kaplan-Meier curve showed that the score was significantly associated with mortality (log-rank *p* < 0.001), as shown in Fig. 3.

Table 3 shows the univariable and multivariable Cox proportional hazard analyses adjusted for age, sex and LVEF (left ventricular ejection fraction) for all-cause death. For patients with calcification score 4–5, the unadjusted hazard ratio (HR) was 4.18 (95% CI: 1.94–9.00, *p* = 0.001) compared to patients with calcification score 0–1. The multivariable Cox proportional hazard analyses showed that calcification score 4–5 was independently associated with all-cause death in STEMI patients after PPCI (HR = 2.32, 95% CI: 1.01–5.31, *p* = 0.046).

**Table 3:** Results of univariable and multivariable Cox proportional hazard analyses for all-cause death

Variables	Univariable analysis			Model 1			Model 2		
	HR	95% CI	*P* value	HR	95% CI	*P* value	HR	95% CI	*P* value
Age									
< 62 years	Reference								
≥ 62 years	4.71	2.09–10.67	**0.001**	3.95	1.48–10.60	**0.006**	3.40	1.40–8.28	**0.007**
Male	0.36	0.19–0.66	**0.001**	0.64	0.33–1.23	0.18	0.65	0.33–1.27	0.207
BMI	0.91	0.82–1.02	**0.093**						
Hypertension	0.72	0.37–1.39	**0.329**						
Diabetes mellitus	1.29	0.68–2.48	**0.438**						
LVEF	0.969	0.947–0.992	**0.009**				0.97	0.95–0.996	**0.019**
Calcification score									
Group 1 (0–1)	Reference								
Group 2 (2–3)	1.66	0.73–3.77	0.236	1.17	0.51–2.72	0.71	0.94	0.40–2.32	0.935
Group 3 (4–5)	4.18	1.94–9.00	**0.001**	2.51	1.12–5.61	**0.025**	2.32	1.01–5.31	**0.046**

Model 1: adjusted for age and male

Model 2: adjusted for age, male, and LVEF

*BMI* body mass index, *CI* confidence interval, *HR* hazard ratio, *LVEF* left ventricular ejection fraction

### The predictive ability of calcification score to 3-year mortality

**Fig. 4:**
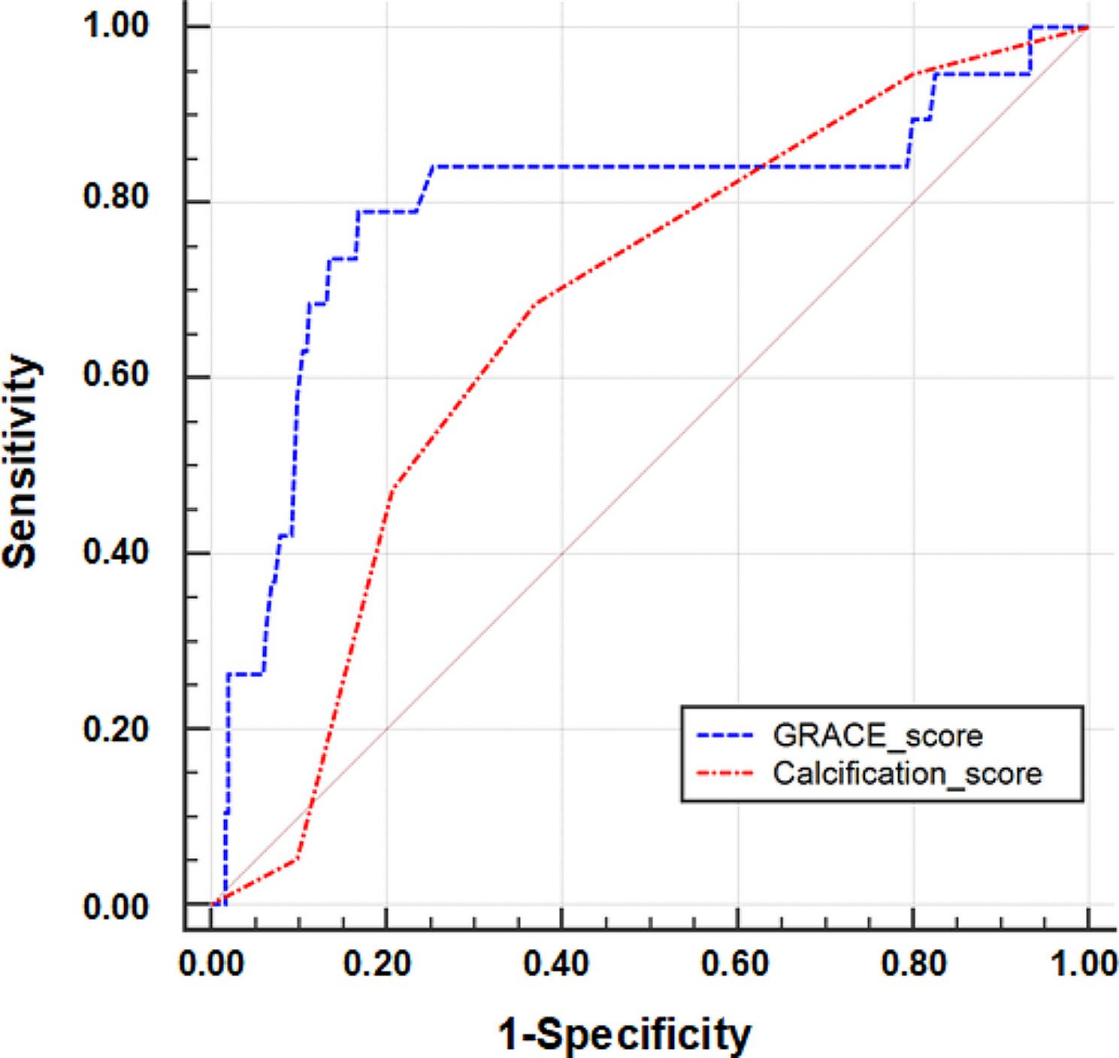
Receiver operating characteristic curves comparing the areas under the curve of calcification score and GRACE score for 3-year mortality. GRACE, Global Registry of Acute Coronary Events

309 (93.1%) patients have the confirmed outcome at 3-year. Figure 4 shows the ROC curves of calcification score and GRACE risk score at 3-year follow-up. The AUC of the calcification score was 0.67 (95% CI: 0.61–0.72), and the AUC of the GRACE score was 0.80 (95% CI: 0.75–0.84). There was no statistical difference in the predictive value between 2 scores for 3-year mortality in STEMI patients after PPCI (*p* = 0.06). Based on the NRI analysis, calcification score showed better risk classification compared with the GRACE score (absolute NRI = 6.63%, *P* = 0.027).

## Discussion

In this retrospective study, we found that the calcification score was independently associated with the long-term prognosis of STEMI patients after PPCI, and that it has certain predictive value for 3-year mortality. This is the first study to estimate the ability of the cumulative calcification score (including CAC, TAC, and AVC) to predict long-term prognosis in a STEMI cohort. The calcification score was easily acquired through existing tests including chest X-ray examination, coronary angiography, and echocardiography, with a similar predictive ability to the GRACE risk score.

Vascular calcification can induce the occurrence and death of cardiovascular diseases. The pathological mechanism may be the adverse effects of calcification on the vascular compliance, vasodilation and plaque stability. Artery wall calcification can occur in intima, media or both. The rigid deposition of calcium minerals in the arterial wall can seriously affect the biomechanics of the arterial system. Increased stiffness can impair vasomotor. Calcification could induce plaque rupture, which is a serious complication of atherosclerosis. Calcification in valves may lead to serious hemodynamic effects, such as severe aortic stenosis. Overall, vascular calcification causes significant clinical adverse effects, including hypertension, left ventricular hypertrophy, coronary heart disease, heart failure, and may lead to plaque rupture, thrombosis, and myocardial infarction [8].

Atheroma plaque calcification is hallmark of advanced atherosclerosis. CAC, TAC, and AVC may have similarities regarding molecular and cellular mechanisms. Regarding the current most accepted theory, the first step is endothelial injury, followed by the release of matrix vesicles upon the macrophage and synthetic vascular smooth muscle cell (VSMC) death which initiates the calcification process of the plaque [9]. After that, matrix metalloproteinases imbalances disrupt extracellular matrix homeostasis and promote leaflet stiffening, while bone morphogenetic protein-2 up-regulates the expression of pro-osteogenic transcription factors. Infiltrated mast cells promote collagen production mediated by valvular interstitial cell, and thus promote the osteogenic interstitial cell differentiation. At last, the secretion of vascular endothelial growth factor exacerbates immune-cell recruitment and cytokine secretion, which in turn boosts the fibro-calcific response [10, 11]. So, it is reasonable to predict adverse events in STEMI patients by using the calcification score combined with CAC, TAC, and AVC.

Vascular calcification and coronary artery disease share similar risk factors. With the increase of calcification score in our research, similar to previous studies [12], patients were older, more likely to have hypertension, dyslipidemia, and diabetes mellitus, and had higher GRACE scores, and higher serum creatinine and BNP levels. On the contrary, with the increase of calcification score in our research, patients had a lower body mass index (BMI), lower proportion of current smoking, and family history of premature coronary heart disease. Consistent with previous research [13], our study also found lesion calcification and its possible association with low BMI. Previous data showed that diabetes was inversely associated with coronary calcium density [14]. However, in our research, the proportion of patients with diabetes was higher in the higher cumulative calcification score group.

The association of vascular and valve calcifications with the clinical outcomes of AMI has been the subject of several studies [2, 5, 15–17]. However, no previous study had explored the relationship between the cumulative calcification score (including CAC, TAC, and AVC) and the prognosis of STEMI. We found that the cumulative calcification score is an independent factor associated with long-term death of STEMI patients after PPCI and has certain predictive value for 3-year all-cause death. As we know, there is no study on the cumulative calcification score. Our study revealed that a calcification score of 4–5 was an independent factor for all-cause death in STEMI patient after PPCI. The ROC curve showed that the AUC of the calcification score was 0.67 (95% CI: 0.61–0.72), which was similar to that of the GRACE risk score (AUC = 0.80, 95% CI: 0.75–0.84). Furthermore, compared with the GRACE risk score, the calcification score was easily acquired through existing test including chest X-ray examination, coronary angiography, and echocardiography, and had similar predictive ability. So, for STEMI patients receiving PPCI, cumulative calcification score maybe a good prognostic predictive tool.

There are several limitations in the present study. First, this was a small, single-center, retrospective study with several inherent limitations, including selection bias. We included consecutive patients during the study period, which might have helped to minimize the bias. Second, computed tomography (CT) can diagnose calcification more accurately; most STEMI patients in our study did not receive CT examination. So, we diagnosed CAC, TAC, and AVC by coronary angiography, chest X-ray, and echocardiography, respectively. Our protocol reflected clinical practice. Third, all patients in this study received percutaneous coronary intervention (PCI) treatment. Therefore, our results may not be applicable to patients receiving coronary artery bypass grafting. Finally, the average age of patients in Group 3 was 72.1 ± 10.1. There were causes of death other than cardiovascular events. In the future, the association of the cumulative calcification score and cardiovascular events needs to be more exploration.

## Conclusion

In this study, we assessed the calcification in LAD, LCX, RCA, thoracic aorta, and aortic valve to calculate the cumulative calcification scores in a cohort of STEMI patients. We found that the calcification score was an independent factor associated with the long-term prognosis of STEMI patients after PPCI, and it has a certain predictive value for 3-year mortality.

## Data Availability

The datasets generated during and/or analyzed during the current study are not publicly available but are available from the corresponding author on reasonable request.

## Abbreviations

CAC: Coronary artery calcification
TAC: Thoracic aortic calcification
AVC: Aortic valve calcification
STEMI: ST segment elevation myocardial infarction
PPCI: Primary percutaneous coronary intervention
LAD: Left anterior descending branch
LCX: Left circumflex branch
RCA: Right coronary artery
ROC: Receiver operating characteristic
GRACE: Global Registry of Acute Coronary Events
CI: Confidence interval
HR: Hazard ratio
AUC: Area under the ROC curve
ECG: Electrocardiogram
SD: Standard deviation
ANOVA: Analysis of variance
BNP: Brain natriuretic peptide
VSMC: Vascular smooth muscle cell
BMI: Body mass index
CT: Computed tomography
PCI: Percutaneous coronary intervention
